# Editorial: Nutritional structures, growth and development processes, and responses to stresses in coarse cereals

**DOI:** 10.3389/fgene.2023.1273932

**Published:** 2023-08-31

**Authors:** Jinbo Li, Yu Fan

**Affiliations:** ^1^ Luoyang Normal University, Luoyang, China; ^2^ Chengdu University, Chengdu, China

**Keywords:** sorghum, low temperature, QTL, aphid, millet, crude protein, maize

This editorial summarizes the contributions to the Frontiers Research Topic “*Nutritional Structures, Growth and Development Processes, and Responses to Stresses in Coarse Cereals*,” established under the Genomics of Plants and the Phytoecosystem, Frontiers in Genetics journals.

Coarse Cereals include maize, sorghum, oats, barley, foxtail millet, rye, buckwheat, Zizania latifolia, Chenopodium quinoa, Amaranthus hypochondriacus, and legumes. As environmentally friendly crops, Coarse crops may have strong environmental adaptability and abiotic stress resistance. For example, broomcorn millet and sorghum is internationally recognized as a strategic reserve crop to cope with a warming climate and an increasingly arid environment (Pardo and VanBuren, 2021). Besides, coarse crops are rich in dietary fiber, vitamins, minerals (especially trace elements), and some antioxidant phytochemicals such as flavonoids, anthraquinones, polyphenols, vitamins, alkaloids, saponins, polysaccharides and peptides. Millet is rich in trace elements selenium, iron and a variety of vitamins, and has the effect of preventing insomnia and cardiovascular and cerebrovascular atherosclerosis; Oat is rich in saponins and β-glucan, which has special effect on reducing blood lipids. Buckwheat is rich in bioflavonoids, which can significantly soften blood vessels and regulate blood lipids and blood sugar (Zhao et al., 2022). Compared to main crops and model plants, research on the formation mechanism, development process, and regulation mechanism of environmental adaptability of special metabolic components of coarse grain crops is insufficient.

This Research Topic aimed to explore recent developments in this area with a focus on the genetic basis of special chemical compositions, nutritional structures, tissue developments, and biological or abiotic adaptability of these coarse grain crops through forward or reverse genetic experiments.

Sorghum is one of the world’s most important cereal crops, an important source of animal feed worldwide and an increasingly important biofuel feedstock. Cold, frost and other low temperature stress seriously affect the agronomic performance of sorghum and limit the geographical distribution of sorghum, which is the main problem of early planting of sorghum in temperate environment. La Borde et al. analyzed the quantitative trait loci of early seed germination and seedling cold tolerance of two sorghum recombinant inbred lines (C1 and C2) by gene sequencing method, constructed linkage maps of 464 and 875 SNPS respectively, and identified 2 and 3 major QTLS for cold resistance, a tool for improving molecular breeding of sorghum germination at low temperature (La Borde et al.).

Sorghum is often attacked by green worms. Huang and Huang used the microarray method to find that 26.1% of the 1,761 cDNA sequences were changed between different treatments of aphid infected sorghum. Meanwhile, 856 DEGs were identified in resistant strains by using RNA-Seq (comparison between 4-day infected and non-infected). Among the resistant genotypes, 4,354 DEGs (4-day resistance genotypes compared with susceptible genotypes) were identified. The two platforms verified each other to screen out multiple common DEGs, indicating that aphids triggered the dynamic defense response of sorghum plants Huang and Huang.

In order to reduce the harm of spider mites to chestnut, Cui et al. screened millet materials with higher crude protein (CP) content in leaves of chestnut interbreeds, and used high-throughput sequencing and identification of genes related to crude protein content in leaves of different millet varieties. 435 differentially expressed genes (DEGs) were identified, and 40 TF genes were identified, which were 11 transcription factor families. It provided important resources to elucidate the accumulation and regulation mechanism of crude protein in millet leaves (Cui et al.).

Maize grown in tropical savannas in West and Central Africa is subject to frequent droughts and Striga infestations, resulting in 30%–100% yield losses. Menkir et al. used mixed model analysis to estimate genetic gains in grain yield and other agronomic traits recorded in an 8-year regional cooperative hybridization trial conducted under managed stress and non-stress conditions and in dryland environments, and found the need to screen inbred flax under both stress conditions. In order to further improve the genetic gain rate of hybrid seed yield in the two stress coexisting areas, the sequential selection scheme has successfully produced hybrids with reliable yield (Menkir et al.).

We are very grateful to several authors for their contributions to this not easy but interesting Research Topic. This Research Topic also conducted a preliminary study on the stress and metabolites of coarse grains, providing a preliminary exploration for future research on coarse grains. We also hope that readers of this Research Topic will find these papers useful.
